# Nootkatone Supplementation Ameliorates Carbon Tetrachloride-Induced Acute Liver Injury via the Inhibition of Oxidative Stress, NF-κB Pathways, and the Activation of Nrf2/HO-1 Pathway

**DOI:** 10.3390/antiox12010194

**Published:** 2023-01-13

**Authors:** Chongshan Dai, Xueyong Zhang, Jiahao Lin, Jianzhong Shen

**Affiliations:** 1 National Key Laboratory of Veterinary Public Health Security, College of Veterinary Medicine, China Agricultural University, Beijing 100193, China; 2 Beijing Key Laboratory of Detection Technology for Animal-Derived Food Safety, China Agricultural University, Beijing 100193, China

**Keywords:** nootkatone, oxidative stress, acute liver injury, Nrf2/HO-1 pathway, NF-κB pathway

## Abstract

Acute liver injury is a type of liver diseases, and it has raised concerns worldwide due to the lack of effective therapies. The aim of this study is to investigate the protective effects of nootkatone (NOOT) on carbon tetrachloride (CCl_4_)-caused acute liver injury in mice. Mice were randomly divided into control, CCl_4_ model, NOOT, and NOOT (5, 10, and 20 mg/kg/day) plus CCl_4_ groups, respectively. Mice in the CCl_4_ plus NOOT groups were orally administrated with NOOT at 5, 10, and 20 mg/kg/days for seven days prior to 0.3% CCl_4_ injection at 10 mL/kg body weight, respectively. Our results showed that NOOT supplementation significantly ameliorated CCl_4_-induced increases of serum AST and ALT levels, hepatocyte necrosis, inflammatory response, oxidative stress, and caspases-9 and -3 activities in the livers of mice. Moreover, NOOT supplementation significantly upregulated the expression of Nrf2 and HO-1 mRNAs but downregulated the expression of NF-κB mRNAs and the levels of IL-1β, IL-6, and TNF-α proteins in the liver tissues, compared to those in the CCl_4_ model group. In conclusion, for the first time, our results reveal that NOOT could offer protective effects against CCl_4_-caused oxidative stress and inflammatory response via the opposite regulation of Nrf2/HO-1 pathway and NF-κB pathway.

## 1. Introduction

In the past 20 years, liver diseases, including acute liver injury, chronic hepatitis, nonalcoholic fatty liver, and liver cancer, have been on the rise. Worldwide, liver diseases have become a leading cause of death [1]. However, so far, it still lacks effective treatment options. Therefore, the development of drugs for treating liver diseases remains an unmet medical need.

Acute liver damage is one class of liver diseases and occurs frequently. It is well known that the induction of oxidative stress, inflammatory response, necrosis, and apoptosis are all considered generally as the basis of acute liver injury caused by drugs, chemicals, or alcohol. These findings also provide the critical targets for clinical treatment and drug development [2]. Carbon tetrachloride (CCl_4_), a highly toxic chemical substance, has been widely used to build an acute liver damage model in rats and mice to develop and identify new hepatoprotective agents [3,4,5]. In the liver tissues of rodents, CCl_4_ could be metabolized quickly to trichloromethyl, dichloromethyl, or monochloromethyl radicals via the metabolic enzyme cytochrome 2E1 (CYP2E1), then producing excessive reactive oxygen species (ROS), and finally inducing cell necrosis or apoptosis [6]. Natural products have various biology functions, including antioxidant, anti-aging, immune-regulation, anti-inflammatory, and anti-microbial activities, which have been considered an important resource for the discovery of new drugs against acute liver damage [3,4,5,7,8,9,10].

**Figure 1:**
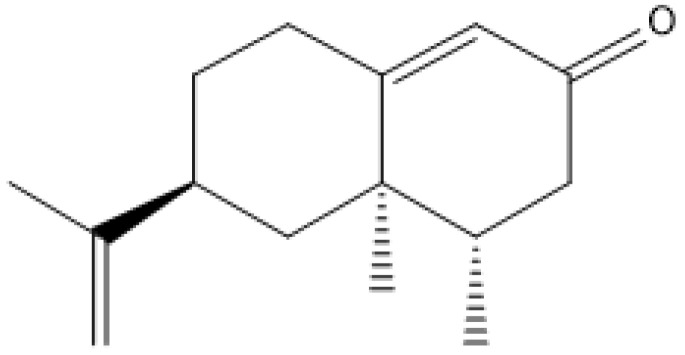
The chemical structure of nootkatone (NOOT).

Many studies have reported that inhibition of oxidative stress or blockade of an inflammatory response may partly contribute to explaining the molecular mechanisms of natural products protecting against acute liver injury caused by CCl_4_ exposure [3,5,11,12]. Nootkatone (NOOT, Figure 1), one of the major active ingredients of the essential oil of *Alpiniae oxyphyllae*, exhibited multiple biological functions, such as antioxidant, anti-inflammatory, neuroprotection, and anti-microbial activities [13,14]. The previous studies have reported that the ethanol extract of *Alpiniae oxyphyllae* supplementation could ameliorate CCl_4_ exposure-caused acute liver injury by upregulating superoxide dismutase (SOD) and catalase (CAT) activities and downregulating malondialdehyde (MDA) levels in vitro [15]. Consistently, a recent study showed that intraperitoneal administration of NOOT at 50 mg/kg per day for four days could effectively inhibit lipopolysaccharide (LPS)-induced oxidative damage and inflammatory response in the brain tissues of mice [14]. NOOT could also directly activate the NF-E2-related factor (Nrf2) signaling pathway, which is a critical endogenous antioxidant signaling pathway in response to oxidative stress and plays a critical role during oxidative damage [9,10,14,15]. In another study, it is also reported that NOOT supplementation could effectively inhibit the expression of NAD(P)H oxidase-4 (NOX4) protein and then inhibit the production of ROS, finally inhibiting cell apoptosis and kidney fibrosis induced by unilateral ureteral obstruction surgical operation in mice [13]. In addition, several studies also reported that NOOT supplementation could directly target the inhibition of the expression of nuclear factor-kappaB (NF-κB), followed by inhibiting the LPS-induced production of pro-inflammatory factors in vitro, such as interleukin-1β (IL-1β), IL-6, TNF-α, and inducible nitric oxide synthase (iNOS) [14].

To date, it is not clear whether NOOT supplementation could provide a protective effect against acute liver injury caused by CCl_4_ exposure. Therefore, in the present study, the protective effects of NOOT supplementation on CCl_4_ exposure-induced acute liver injury were explored by using a mouse model. Furthermore, the underlying molecular mechanisms involving the regulation of Nrf2 and NF-κB pathways were explored.

## 2. Materials and Methods

### 2.1. Chemicals and Reagents

NOOT (CAS number: 4674-50-4 and its purity ≥ 97%) was provided by Aladdin Reagent Company (Shanghai, China). CCl_4_ was provided by Kaixing Chemical Company (Tianjin, China). BCA™ protein assay kit was obtained from Beyotime company (Haimen, China). Carboxyl methyl cellulose sodium (CMC-Na) was purchased from Sigma-Aldrich (Shanghai, China).

### 2.2. Animals and Treatments

In the present study, the Institutional Animal Care and Use Committee of China Agricultural University approved the animal experiments at 1 June 2022 (approvement number: CAU20220601-1). C57BL/6 mice aged 8-week-old (male; the body weight was in the range of 20–22 g) were obtained from Vital River Animal Technology Company (Beijing, China). Before the treatments, all mice were given adaptive feeding for one week. Five mice were kept in each cage and all mice had free access to water and food during experiments and in a standard animal house with the room temperature at 22 ± 3 °C and the relative humidity at 55 ± 5%. A 12 h light–dark cycle was performed during experiments.

All mice were randomly divided into six groups: untreated control group, NOOT 20 mg/kg/day group (i.e., NOOT 20 group), CCl_4_ model group, NOOT 5 mg/kg/day plus CCl_4_ group (i.e., CCl_4_+NOOT 5 group), NOOT 10 mg/kg/day plus CCl_4_ group (i.e., CCl_4_+NOOT 10 group), and NOOT 20 mg/kg/day plus CCl_4_ group (i.e., CCl_4_+NOOT 20 group). Eight mice were in each group. All mice in the CCl_4_-treated groups were injected intraperitoneally (i.p.) with 0.3% CCl_4_ (it was dissolved in olive oil) at the dose of 10 mL/kg body weight. NOOT was suspended using 0.5% CMC-Na to the final working solution for standby. Mice in the NOOT-treated groups were treated with the final concentrations of 5, 10, and 20 mg/kg/day for seven days prior to CCl_4_ treatment. Mice in the NOOT 20 mg/kg/day group were orally treated with NOOT at the dose of 20 mg/kg per day for seven days. In the control group, mice were orally treated with an equal volume of vehicle (i.e., 0.5% CMC-Na) and injected i.p. with an equal volume of olive oil. At 24 h after CCl_4_ administration, mice were anesthetized for euthanasia using the i.p. injection of pentobarbital sodium at 80 mg/kg body weight. The blood and liver samples were isolated for the corresponding histopathological and biochemical measurements.

### 2.3. Serum Biochemical Analysis

Blood samples of mice were collected in 1.5 mL Eppendorf tubes, and then the blood samples were centrifuged at 3000× *g* for 15 min, and the serum samples were collected for the biochemical measurements. The activities of aspartate aminotransferase (AST) and alanine aminotransferase (ALT) in the serum were determined by using a Hitachi 7080 automatic analyzer (Hitachi High-Technologies Corporation Company, Japan) according to the a previously published study [3].

### 2.4. Histopathological Assessment

The liver tissues from four mice in each group were fixed in 4% neutral formaldehyde for the histopathological analysis. The liver tissues were treated with a series of gradient ethanol dehydration, transparency, embedding, and section. Hematoxylin-eosin staining was performed according to a previous study [3]. Furthermore, the degree of liver injury was scored using a semi-quantitative score (SQS) system, according to the previous study [3]. The detail information of SQS was shown in Supplementary File S1.

### 2.5. Measurement for Malondialdehyde (MDA) Levels, and Catalase (CAT), and Superoxide Dismutase (SOD) Activities in the Liver Tissues of Mice

Parts of liver tissues (about 50 mg) were isolated and homogenized with 0.5 mL of cold Tris-buffer. Then, the homogenates were collected and centrifuged at 3000× *g* for 15 min at a temperature of 4 °C. Then, the supernatants were collected for the measurements of MDA levels and CAT and SOD activities, according to the corresponding manufacturer’s instructions provided by the MDA, CAT, and SOD commercial kits (Nanjing Jiancheng Company, Nanjing, China), respectively. A BCA™ protein assay kit was employed to measure the protein concentration of each sample.

### 2.6. Measurement of Inflammatory Markers IL-1β, TNF-α, and IL-6 Levels in Liver Tissues of Mice

The levels of inflammatory markers, including TNF-α, IL-1β, and IL-6 proteins, were determined using the corresponding TNF-a, IL-1 β and IL-6 ELISA kits (R&D Systems, Minneapolis, MN, USA). A BCA™ protein assay kit was employed to measure the protein concentration of each sample.

### 2.7. Measurements of Caspases-9 and -3 Activities in the Liver Tissues of Mice

A small part of liver tissues (about 20 mg) was isolated and lysed at 4 °C using 0.5 mL lysis buffer provided by the commercial kits for 15 min. Then, the lysate of each sample was centrifuged at 12,000× *g* for 15 min. After centrifugation, the supernatants were collected for the measurement of caspases-3 and -9 activities using the commercial caspases-3 and -9 kits (Beyotime Company, Haimen, China). A BCA™ protein assay kit was employed to measure the protein concentration of each sample.

### 2.8. Quantitative Reverse-Transcription (qRT)-PCR for the Gene Expression

About 20 mg liver tissues were used to isolate the total RNA for the gene expression analysis. Briefly, the total RNA isolations were performed by using a FastPure Cell/Tissue Total RNA Isolation Kit (No. RC112-01, Vazyme Biotech Co., Ltd., Nanjing, China). The quality of RNAs was assessed using a Nanodrop reader (Therma Fisher, Waltham, MA, USA) and the values of the optical density at 260/280 nm among 1.9~2.1. 500 ng of total RNAs were used to synthesize the cDNAs using a Prime Script RT-PCR kit (Takara Company, Beijing, China). All the primer sequences are shown in Supplementary Table S1. β-actin was used as the internal control gene. An AB7500 real-time PCR instrument (Applied Biosystems, Waltham, MA, USA) was used to perform the qRT–PCR. The 2^−ΔΔCt^ method was employed to calculate the relative expression of targeted genes.

### 2.9. Statistical Analysis

In the present study, all data are analyzed and shown as mean ± standard deviation (S.D.) unless the specifical mention. A one-way analysis of variance (ANOVA) was selected for the statistical analysis. Then, Tukey’s multiple comparisons post hoc test was further performed. A *p*-value < 0.05 was considered statistically significant.

## 3. Results

### 3.1. NOOT Supplementation Ameliorates CCl_4_-Induced Liver Dysfunction in Mice

**Figure 2:**
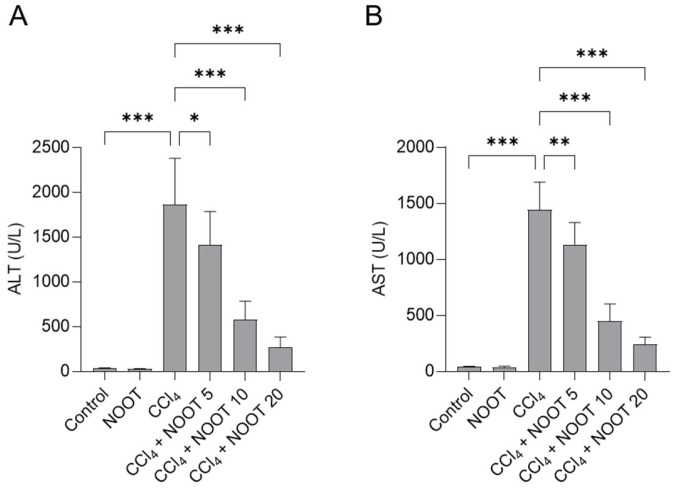
The changes in serum ALT (**A**) and AST (**B**) levels. All results are shown as mean ± S.D. (*n* = 8). * *p* < 0.05, ** *p* < 0.01, and *** *p* < 0.001, compared between two different groups.

During the course of the experiment, no mice died. The liver functions of mice were further assessed. As shown in Figure 2, in the CCl_4_-treated mice, the levels of serum ALT and ALT markedly increased to 1863.8 U/L and 1443.0 U/L (both *p* < 0.001), respectively, compared to those in the control group. NOOT pre-treatment significantly improved CCl_4_ exposure-induced liver dysfunction. NOOT supplementation at the doses of 10 and 20 mg/kg/day for 7 days significantly decreased the levels of serum ALT to 577.3 U/L and 281.9 U/L (both *p* < 0.001), respectively, and significantly decreased the levels of serum AST to 451.4 U/L and 242.4 U/L (both *p* < 0.001), respectively, compared to those in the CCl_4_ model group. NOOT-alone treatment did not change the levels of serum ALT and AST, compared to those in the control group (Figure 2).

### 3.2. NOOT Supplementation Ameliorates the Acute Liver Injury Caused by CCl4 Exposure

**Figure 3:**
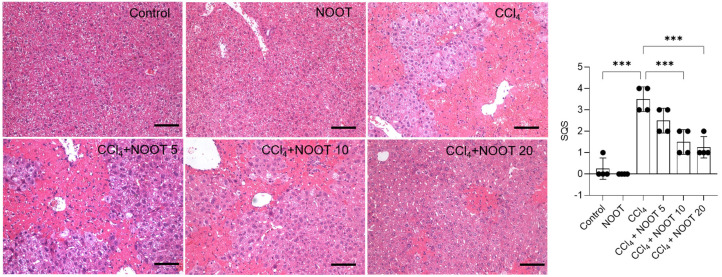
NOOT supplementation ameliorates CCl_4_-induced histopathological changes in the liver tissues of mice. Representative histopathological changes (on the **left**) and the corresponding the semi-quantitative score (SQS) were shown (on the **right**). Data are shown as mean ± S.D. (*n* = 4). *** *p* < 0.001, compared between two different groups. NOOT, nootkatone. Bar = 50 μm.

Compared to the control, CCl_4_ treatment resulted in marked histopathological injury in the liver tissues, which was significantly ameliorated by NOOT pretreatment in a dose-dependent manner. As shown in Figure 3, CCl_4_ treatment caused cell necrosis, and inflammatory cell infiltrations in the liver tissues and the corresponding SQS increased to 3.5 (*p* < 0.001), compared to that in the control group. NOOT supplementation could effectively improve CCl_4_ exposure-caused histopathological injuries. In the CCl_4_ + NOOT 10, and CCl_4_ + NOOT 20 groups, the SQS decreased to 1.5, and 1.0 (both *p* < 0.001), respectively. There was no marked histopathological change in the NOOT-alone treatment group compared to that in the control group.

### 3.3. NOOT Supplementation Ameliorates CCl_4_ Exposure-Caused Oxidative Stress

**Figure 4:**
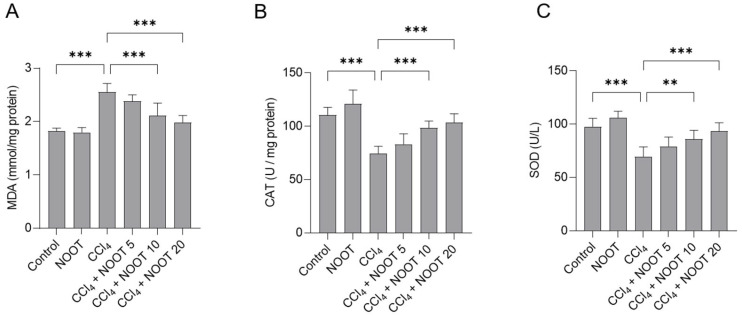
NOOT supplementation ameliorates CCl_4_ exposure-caused oxidative stress in the liver tissues of mice. Mice were orally pretreated with NOOT at the doses of 5, 10, and 20 mg/kg/day for 7 days prior to CCl_4_ exposure, then, the levels of MDA (**A**), and the activities of CAT (**B**) and SOD (**C**) in the liver tissues of mice were measured. Data are presented as mean ± S.D. (*n *= 8). ** *p* < 0.01, and *** *p* < 0.001, compared between two different groups. NOOT, nootkatone.

The biomarkers of oxidative stress, including the levels of MDA, and the activities of CAT, and SOD were determined. As shown in Figure 4, CCl_4_ treatment significantly increased the levels of MDA to 2.56 mmol/mg protein and significantly decreased the activities of CAT and SOD to 74.5 U/mg protein and 69.6 U/mg protein (all *p* < 0.001), respectively. NOOT supplementation significantly ameliorated CCl_4_ exposure-induced oxidative stress damage in the liver tissues. Correspondingly, in the CCl_4_ + NOOT 10, and CCl_4_ + NOOT 20 groups, MDA levels decreased to 2.11 mmol/mg protein and 1.98 mmol/mg protein, respectively; CAT activities increased to 98.4 U/mg protein and 103.3 U/mg protein, respectively; SOD activities increased to 85.9 U/mg protein and 93.3 U/mg protein, respectively. Compared to the control, mild increases in the levels of CAT and SOD activities and mild decreases in the MDA levels were detected in the NOOT-alone treatment group (Figure 4).

### 3.4. NOOT Supplementation Ameliorates CCl4-Induced Inflammtory Response

**Figure 5:**
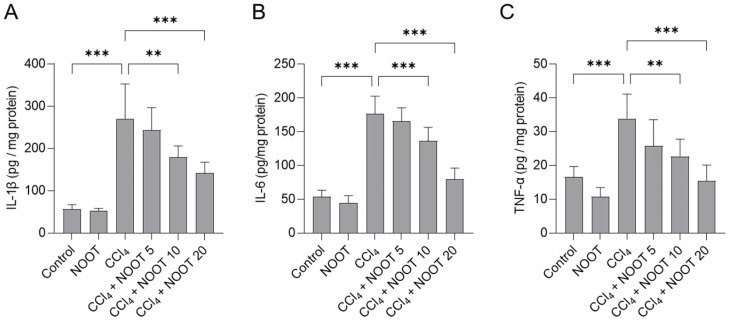
NOOT supplementation ameliorates CCl_4_ exposure-caused inflammatory response in the liver tissues of mice. Mice were orally pretreated with NOOT at the doses of 5, 10, and 20 mg/kg/day for 7 days prior to CCl_4_ exposure; then, the levels of IL-1β (**A**), IL-6 (**B**), and TNF-α (**C**) proteins in the liver tissues of mice were measured. Data are shown as mean ± S.D. (*n* = 8). ** *p* < 0.01, and *** *p* < 0.001, compared between two different groups. NOOT, nootkatone.

As shown in Figure 5, CCl_4_ exposure significantly increased the levels of IL-1β, IL-6, and TNF-α proteins in the liver tissues of mice. Compared to the CCl_4_ model group, NOOT supplementation at 10 and 20 mg/kg/day for 7 days significantly decreased the levels of IL-1β protein from 270.9 pg/mg protein to 179.7 pg/mg protein, and 142.1 pg/mg protein (*p* < 0.01 or *p* < 0.001), respectively; significantly decreased the levels of IL-6 protein from 176.5 pg/mg protein to 136.3 pg/mg protein, and 80.1 pg/mg protein (both *p* < 0.001), respectively; and significantly decreased the levels of TNF-α protein from 33.9 pg/mg protein to 22.7 pg/mg protein, and 15.4 pg/mg protein (*p* < 0.01 or *p* < 0.001), respectively. These inflammatory markers had no marked changes in the liver tissues of mice in the NOOT-alone group, compared to that in the control group.

### 3.5. NOOT Supplementation Ameliorates CCl_4_ Exposure-Caused the Activation of Caspases-9 and -3

**Figure 6:**
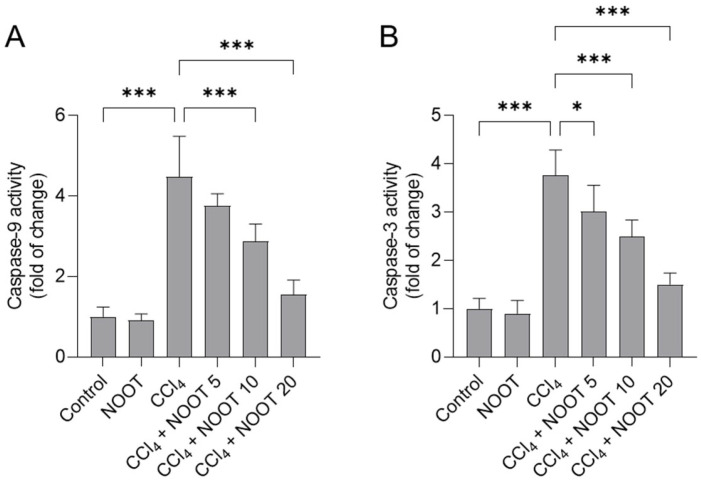
NOOT supplementation ameliorated CCl_4_ exposure-caused the activation of caspases-9 and -3 in the liver tissues of mice. Mice were orally pretreated with NOOT at the doses of 5, 10, and 20 mg/kg/day for 7 days prior to CCl_4_ exposure; then, the activities of caspases-9 (**A**) and -3 (**B**) in the liver tissues of mice were measured. Data are shown as mean ± S.D. (*n* = 6). * *p <* 0.05, and *** *p* < 0.001, compared between two different groups. NOOT, nootkatone.

Compared to the control group, CCl_4_ exposure significantly upregulated the levels of caspases-9 and -3 activities to 4.49- and 3.76-fold (both *p <* 0.001) in the liver tissues of mice, respectively (Figure 6). Compared to the CCl_4_ model group, NOOT supplementation at the doses of 10 and 20 mg/kg/day for 7 days significantly decreased caspase-9 activities to 2.87- and 1.55-fold (both *p <* 0.001), respectively, and significantly decreased caspase-3 activities to 2.49- and 1.50-fold (both *p <* 0.001), respectively. There was no marked change in the levels of caspases-9 and -3 in the liver tissues of mice in the NOOT-alone treatment group, compared to those in the control group.

### 3.6. NOOT Supplementation Downregulates the Expression of NF-κB mRNA and Upregulates the Expression of Nrf2 and HO-1 mRNAs

**Figure 7:**
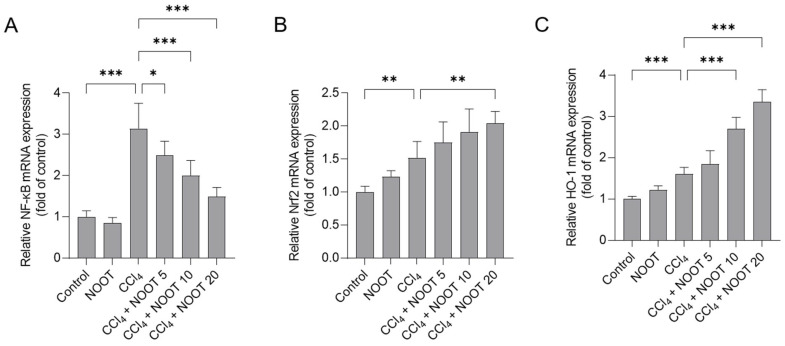
The relative expression of NF-κB (**A**), Nrf2 (**B**), and HO-1 (**C**) mRNAs in the liver tissues of mice. Data are shown as mean ± S.D. (*n*  =  6). * *p <* 0.05, ** *p <* 0.01, and *** *p* < 0.001, compared between two different groups. NOOT, nootkatone.

The relative expressions of NF-κB, Nrf2, and HO-1 mRNAs in the liver tissues of mice were examined. Compared to the untreated control group, CCl_4_ exposure significantly upregulated the expression of NF-κB, Nrf2, and HO-1 mRNAs to 3.14-, 1.54-, and 1.61-fold (*p <* 0.01 or *p <* 0.001), respectively (Figure 7). NOOT supplementation could regulate the relative expression of these genes. As shown in Figure 7, in the CCl_4_+ NOOT 10 and CCl_4_+ NOOT 20 groups, the relative expression of NF-κB mRNA decreased to 2.01- and 1.49-fold, respectively (both *p <* 0.001), while the relative expression of Nrf2 mRNA increased to 1.84-, and 2.23-fold (*p <* 0.01), respectively, and the relative expression of HO-1 mRNA decreased 2.69-, and 3.35-fold (both *p <* 0.001), respectively, compared to the CCl_4_ model group.

## 4. Discussion

Chemical liver injury is one class of acute liver injury and liver diseases have become a global public health burden [1]. It has been reported that many hazardous substances, including aflatoxin B1, cadmium, and mercury chloride, could induce acute liver injury, finally resulting in acute hepatitis or liver failure in people [16,17,18]. Currently, effective drugs for treating and preventing acute liver injury are still scarce, and it is essential for the development of effective therapeutic agents against acute liver injury induced by environmental hazardous substances. In this study, our results found that NOOT, a natural product isolated from *Alpinia oxyphylla*, could effectively ameliorate CCl_4_ exposure-caused acute liver dysfunction in a mouse model (Figure 2, Figure 3, Figure 4, Figure 5, Figure 6 and Figure 7). The potential molecular mechanisms may involve antioxidant and anti-inflammatory activities through the activation of the Nrf2/HO-1 pathway and the inhibition of the NF-κB pathway (Figure 2, Figure 3, Figure 4, Figure 5, Figure 6 and Figure 7).

CCl_4_ is a common hepatotoxic compound. It is usually used to establish the acute liver injury model for the development of effective hepatoprotectants in rodents [5,19]. In line with the previous studies [3,4,20], our data showed that CCl_4_ exposure significantly increased the levels of serum ALT and AST by about 40~50-fold (Figure 2), indicating that acute liver dysfunction model in mice was successfully established. NOOT supplementation at 5, 10, and 20 mg/kg/days for 7 days could dose-dependently decrease the levels of serum ALT and AST (Figure 2), as well as the histopathology damage (Figure 3), indicating the hepaprotective effects of NOOT. Very recently, Yan et al. reported that oral supplementation of NOOT at the doses of 5 or 10 mg/kg/day for 4 weeks could partly abolish d-galactosamine-induced acute liver injury in mice [21]. These data indicated that NOOT supplementation could provide a protective effect against liver dysfunction caused by CCl_4_ exposure.

It is well known that excessive ROS production is the main basis of CCl_4_ exposure-induced liver and renal injuries [3,4,20,22]. Excessive ROS production can directly damage DNA, lipids, proteins, and the main organelles of cells, such as the mitochondrion, endoplasmic reticulum, and lysosome [4,10,16,23]. A previous study reported that CCl_4_ exposure could significantly promote the production of ROS in liver tissues and induce liver dysfunction in mice [24]. In the current study, our data showed that CCl_4_ treatment significantly upregulates the levels of MDA and downregulates the activities of SOD and CAT in the liver tissues of mice. NOOT supplementation effectively inhibited CCl_4_-induced increases in MDA levels and rescued the levels of SOD and CAT in the liver tissues of mice (Figure 4). MDA is a biomarker of lipid peroxidation [25]. SOD and CAT are considered two important antioxidant enzymes, which can directly catalyze superoxide anion and hydrogen peroxide (H_2_O_2_), respectively, and both finally form non-toxic substances in cells [26]. NOOT was shown to have potent antioxidant activity and NOOT supplementation can effectively inhibit the production of ROS, reduce MDA levels, and upregulate the activities of SOD and CAT in H_2_O_2_-treated PC12 cells in vitro [27]. It was also reported that NOOT supplementation could protect against doxorubicin-induced cardiotoxicity and induced testicular toxicity induced by water pipe smoke in mice by inhibiting lipid peroxidation and upregulating the activities of SOD and CAT [28,29]. Taken together, our data indicated that NOOT co-administration could provide a protective effect via the upregulation of endogenous antioxidant enzymes SOD and CAT activities.

In the process of liver dysfunction caused by CCl_4_ exposure, a concurrent inflammatory reaction was usually induced [30,31,32,33,34,35,36,37,38]. In this study, marked inflammatory responses in the CCl_4_-treated liver tissues were detected, which were evident due to the increased expression of IL-1β, IL-6, and TNF-α proteins, as well as the inflammatory cell infiltrations in the liver tissues of mice (Figure 5). These findings are consistent with the previous literature [3,4,20,22,30,31,32,33,34,35,36,37,38]. In addition, our data showed that NOOT supplementation could effectively inhibit the expression of IL-1β, IL-6, and TNF-α proteins induced by CCl_4_ exposure (Figure 5). Several animal and in vitro cell experiments also found that NOOT supplementation could significantly inhibit LPS-, carrageenan-, anterior-cruciate-ligament-transection-, and isoproterenol-induced acute or chronic inflammatory responses [14,39,40,41]. Taken together, these evidences indicate that NOOT supplementation may provide hepaprotective effects by inhibiting the inflammatory response. Moreover, previous studies have demonstrated that the inhibition of NF-κB, adenosine 5‘-monophosphate (AMP)-activated protein kinase (AMPK), or Toll-like receptor 4 (TLR4) pathways could partly contribute to explaining the anti-inflammatory mechanisms of NOOT [14,40,41]. NF-κB is a vital transcriptional factor that could activate the expression of number of pro-inflammatory cytokines, such as TNF-α, IL-1β, and IL-6 [42]. In this study, our results showed that NOOT supplementation can significantly inhibit CCl_4_ exposure-caused the expression of NF-κB mRNA in the liver tissues (Figure 7). Therefore, these evidences indicate that the anti-inflammatory effect of NOOT may be partly attributed to the inhibition of the NF-κB pathway. Furthermore, a recent study found that NAD(P)H quinone oxidoreductase 1 (NQO1) knockdown could partly attenuate the anti-inflammatory effects of NOOT in LPS-treated cells [14]. Therefore, there is a cross-talk between Nrf2 and NF-κB pathways in the protective effects of NOOT against CCl_4_ exposure-caused liver dysfunction. Therefore, the precise actional mechanisms need more studies.

Apoptosis is a type of programmed cell death in mammalian cells. It can be activated by many signaling pathways [43]. In the present study, significant upregulation of caspases-9 and -3 activities in CCl_4_-treated liver tissues of mice were detected, which were partly revised by NOOT supplementation in a dose-dependent manner (Figure 6). It is well known that caspase-3 is a classical biomarker of apoptosis and caspase-9 is a critical biomarker in the process of the mitochondrial apoptotic pathway [43]. Previous studies have shown that CCl_4_ exposure could cause mitochondrial dysfunction, resulting in inducing the expression of cytochrome C (CytC), caspase-9, and caspase-3, finally inducing apoptotic cell death [3,4,11,44]. Consistently, Nemmar et al. found that NOOT supplementation at 90 mg/kg per day could significantly inhibit caspase-3 activation and DNA damage induced by diesel exhaust particles [45]. In another study, the authors found that *Alpiniae oxyphyllae* extract (protocatechuic acid and NOOT are suggested as the two main components) supplementation could effectively inhibit mitochondrial dysfunction, and caspase-3 activation, and apoptotic cell death induced by H_2_O_2_ in PC12 cells [27]. In short, this evidence indicates that NOOT supplementation can improve liver dysfunction caused by CCl_4_ exposure via the inhibitory effect on the mitochondrial apoptotic pathway.

Recently, several studies have reported that NOOT is the activator of Nrf2, a vital transcription factor in response to oxidative stress [14,21,41]. Under the normal condition, Nrf2 is located in the cytoplasm via the interaction with Kelch-like ECH-associated protein 1 (Keap1), which results in the ubiquitin-dependent degradation of Nrf2. Under the stress condition, Nrf2 is separated from Nrf2-Keap1 complex and enters the nucleus; then, it transcriptionally upregulates the expression of more than 200 cell protective genes, such as HO-1, SOD, CAT, and NQO1 [46]. In our previous studies, it was shown that the knockdown of Nrf2 or pharmacology inhibition of HO-1 could exacerbate the cytotoxicity in CCl_4_ -treated HepG2 cells or acute liver injury in mice [3,11]. In the present study, our data showed that NOOT supplementation could significantly upregulate the mRNA expression of Nrf2 and its downstream gene HO-1 (Figure 7). Consistently, Meeran et al.’s study found that NOOT cotreatment at 10 mg/kg/day for 10 days could effectively upregulate the expression of Nrf2 and HO-1 proteins in heart tissues and protect against isoproterenol-induced heart toxicity in rats [41]. Park et al. showed that NOOT treatment could significantly activate the transcriptional expression of Nrf2 and the expression of HO-1 and NQO1 proteins in LPS-treated BV2 cells [14]. Furthermore, the pharmacological inhibition or genic silencing of NQO1, a key downstream target of Nrf2, partly attenuated the inhibitory effects of NOOT on LPS-induced ROS production in BV2 cells [14]. In short, these evidences indicate that the activation of Nrf2/HO-1 may partly contribute to the protective effect of NOOT against liver injury caused by. In addition, the activation of Nrf2 pathway may also partly support the antioxidant activities of NOOT via the transcriptional upregulation of SOD and CAT genes. However, the precise mechanisms still require more investigation.

*Alpiniae oxyphyllae*, one of the “four famous south medicines” in China, has been widely used as a food and medicine in China [47]. As a traditional Chinese medicine, *Alpiniae oxyphyllae* has been used for treating diarrhea and neurodegenerative diseases [47]. NOOT is one of the main active ingredients. Like *Alpiniae oxyphyllae*, the therapeutic effects of NOOT on liver disease, kidney disease, and neurodegenerative disease have been illustrated in vitro and animal studies [47,48]. However, the information on the safety and pharmacokinetic parameters of NOOT is still limited, so it should be further investigated before being applied to humans.

## 5. Conclusions

**Figure 8:**
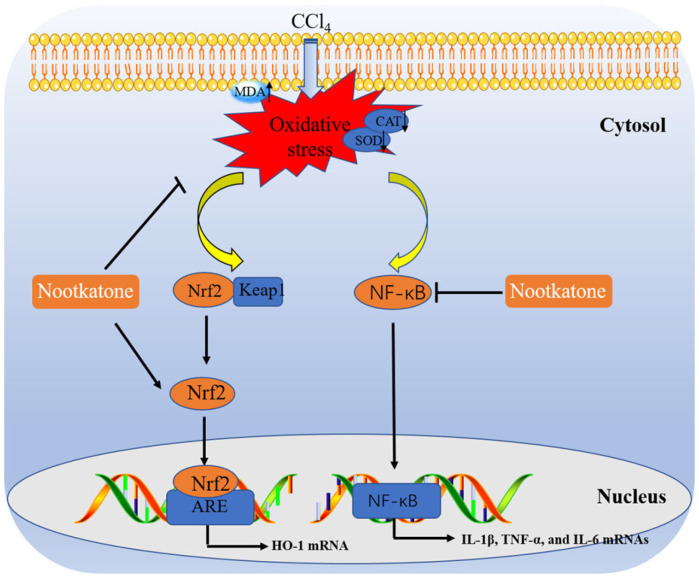
A proposed work model of NOOT protecting against acute liver injury caused by CCl_4_ exposure. MDA, malondialdehyde; CAT, catalase; SOD, superoxide dismutase; Nrf2, NF-E2-related factor; NF-κB, nuclear factor-kappaB; IL-1β, interleukin-1β; IL-6, interleukin-6; TNF-α, tumor necrosis factor-α; HO-1, heme oxygenase-1; ARE, antioxidant response element.

In conclusion, for the first time, our data reveal that NOOT supplementation can effectively improve CCl_4_ -induced acute liver injury through inhibiting lipid peroxidation, oxidative stress, and inflammatory response, which may be partly attributed to the activation of Nrf2/HO-1 pathway and the inhibition of NF-κB pathway. A proposed work model of NOOT protecting against acute liver injury induced by CCl_4_ exposure was shown in Figure 8. Our current study shed that NOOT may be a promising candidate for treating acute liver injury.

## Data Availability

The data are contained within this article.

## Supplementary Materials

The following supporting information can be downloaded at https://www.mdpi.com/article/10.3390/antiox12010194/s1. Supplementary File S1: Histopathological assessments; Table S1: the primer information for qRT-PCR.
