# Supplementary material for: Nootkatone Supplementation Ameliorates Carbon Tetrachloride-Induced Acute Liver Injury via the Inhibition of Oxidative Stress, NF-κB Pathways, and the Activation of Nrf2/HO-1 Pathway
Source: Antioxidants (Basel). 2023 Jan 13;12(1):194. doi: 10.3390/antiox12010194 (PMC9855146; doi:10.3390/antiox12010194)
Supplement: Supplementary file 1 [file antioxidants-12-00194-s001.zip › antioxidants-2159632-supplementary.pdf]

Supplementary File S1. Histopathological assessments

The liver tissues from four mice in each group were fixed in 4% neutral formaldehyde for the histopathological analysis. The liver tissues were treated with a series of gradient ethanol dehydration, transparency, embedding, and section, and hematoxylin-eosin staining was performed according to the previous study [3]. Furthermore, the degree of liver injury was scored using a semi-quantitative score (SQS) system, according to the previous study [3], as follows: Grade 0 showed no marked pathological change. Grade 1 showed the presence of hepatocyte degeneration with small rare of foci of necrosis. Grade 2 showed the small area of mild centrilobular necrosis around the central vein. Grade 3 showed the area of mild centrilobular necrosis that is severer than Grade 2. Grade 4 showed the centrilobular necrosis that is severer than Grade 3.

Supplementary Table S1. Primer sequences of the quantitative real-time PCR.

Gene name	Gene accession number	Direction	Primer sequence (5′to 3′)
NF-kB	NM_008689	forward	5′- GCTGCCAAAGAAGGACACGACA -3′
		reverse	5′-GGCAGGCTATTGCTCATCACAG -3′
Nrf2	NM_010902	forward	5′- CAGCATAGAGCAGGACATGGAG -3′
		reverse	5′- GAACAGCGGTAGTATCAGCCAG -3′
HO-1	NM_010442	forward	5′- CACTCTGGAGATGACACCTGAG -3′
		reverse	5′- GTGTTCCTCTGTCAGCATCACC -3′

β-actin	NM_007393	forward	5′- CATTGCTGACAGGATGCAGAAGG -3′
		reverse	5′- TGCTGGAAGGTGGACAGTGAGG -3′
